# The D-Dimer/Albumin Ratio Is a Prognostic Marker for Aneurysmal Subarachnoid Hemorrhage

**DOI:** 10.3390/brainsci12121700

**Published:** 2022-12-12

**Authors:** Wei Wu, Xunzhi Liu, Qi Zhu, Xiangxin Chen, Bin Sheng, Jiatong Zhang, Wei Li, Dingding Zhang, Chunhua Hang

**Affiliations:** 1 Department of Neurosurgery, Nanjing Drum Tower Hospital Clinical College of Xuzhou Medical University, Xuzhou 221004, China; 2 Department of Neurosurgery, Nanjing Drum Tower Hospital, The Affiliated Hospital of Nanjing University Medical School, Nanjing 210008, China; 3 Department of Neurosurgery, Nanjing Drum Tower Hospital, Clinical College of Nanjing Medical University, Nanjing 210008, China

**Keywords:** aneurysmal subarachnoid hemorrhage, D-dimer/albumin ratio, disease severity, outcomes

## Abstract

**Background**: Aneurysmal subarachnoid hemorrhage (aSAH) is a severe neurological event with limited treatment options, and little is known about its pathophysiology. There are few objective tools for predicting outcomes of aSAH patients and further aiding in directing clinical therapeutic programs. This study aimed to determine whether an elevated serum D-dimer/albumin ratio (DAR) reflects disease severity and predicts aSAH outcomes. **Methods**: We included 178 patients with aSAH. Data included demographics; clinical severity of aSAH (World Federation of Neurological Societies (WFNS) grade and Hunt–Hess grade); levels of D-dimer, albumin, and c-reactive protein (CRP); leukocyte counts on admission; and three-month outcomes. The outcomes were dichotomized into good and poor. The predictive ability of DAR for outcomes was determined using receiver operating characteristic (ROC) curve analysis. **Results**: Serum DAR showed a positive correlation with disease severity. Univariate analysis revealed that DAR, WFNS grade, Hunt–Hess grade, delayed cerebral infarction (DCI), age, neutrophil-to-lymphocyte ratio (NLR), and CRP/albumin ratio (CAR) were associated with unfavorable outcomes. Multivariate regression analysis further revealed that elevated DAR predicted poor outcomes after adjusting for WFNS grade, Hunt–Hess grade, DCI, age, NLR, and CRP/albumin ratio. Receiver operating characteristic curve analysis revealed that DAR predicted outcomes at a level comparable with NLR and CAR and had superior predictivity than D-dimer alone. **Conclusion**: DAR is a promising objective tool for aSAH outcome prediction. A high content DAR was associated with disease severity and unfavorable short-term outcomes.

## 1. Introduction

Aneurysmal subarachnoid hemorrhage (aSAH) is a devastating neurological disorder with significant high mortality and morbidity [1]. A limited understanding of the pathophysiology has hampered the development of effective therapeutics. The scarcity of treatment options warrants novel methods for evaluating the severity of aSAH and possible therapeutic candidates. Many classification systems evaluate the severity and outcomes of aSAH upon hospital admission, including the World Federation of Neurological Societies (WFNS) grade and the Hunt–Hess grade (H-H). These rating scales are primarily based on subjective judgments from medical staff, creating bias, and they have limited value in generating prognoses [2]. An objective indicator is needed to assess the severity of aSAH and assist in outcome prediction. Several blood tests might help predict outcomes [3], including tests for D-dimer, where high levels indicated poor outcomes in aSAH, and albumin, where low levels have been associated with poor aSAH outcomes [4,5]. The D-dimer/albumin ratio (DAR) has been identified as an independent predictor of poor outcome in patients suffering from infection, malignancy and critical illness [6,7]. However, the prognostic value of the combination of D-dimer and albumin has not been evaluated in aSAH patients. Therefore, this study aimed to validate the prognostic ability of DAR in the clinical outcomes of aSAH patients.

## 2. Methods

### 2.1. Patients

This was a single-center, retrospective study of all patients with aSAH admitted to the Department of Neurosurgery, Nanjing Drum Tower Hospital, the Affiliated Hospital of Nanjing University Medical School, from March 2020 to December 2021. The inclusion criteria were as follows: ≥18 years; admission within 24 h of aSAH onset; Serum D-dimer and albumin measurements completed upon hospital admission. The exclusion criteria were: subarachnoid hemorrhage associated with autoimmune disease, inflammatory disease, liver disease, renal impairment, malnutrition, trauma, arteriovenous malformation, or moyamoya disease. The selection of appropriate treatment modality (clipping or coiling) was consistent with current guidelines [8,9]. The Medical Ethics Committee of the Affiliated Nanjing Drum Tower Hospital approved the study. All methods were performed following relevant guidelines.

### 2.2. Data Collection

Data collected included the patient demographics, baseline characteristics at hospital admission, and clinical outcomes of patients. The patient demographics included age, sex, and medical history. We diagnosed aSAH at admission using computed tomography (CT) or digital subtraction angiography (DSA). The presentation of aSAH was evaluated using the WFNS grade, H-H grade [10], and modified Rankin Scale (mRS) scores. The occurrence of acute hydrocephalus and delayed cerebral ischemia (DCI) was determined by clinical presentation and radiological examination. Serum levels of D-dimer, c-reactive protein (CRP), albumin, neutrophils, and lymphocytes were recorded. DAR, CRP/albumin ratio (CAR), and neutrophil/lymphocyte ratio (NLR) were calculated. At a follow-up time of 3 months, patient outcomes were assessed by mRS scores, with mRS scores of 0–2 regarded as a good outcome and mRS scores of 3–6 regarded as a poor outcome [11].

### 2.3. Statistical Analysis

We dichotomized patients into two groups (good and poor outcomes) to assess the prognostic potential of DAR and other factors based on the 3-month follow-up mRS scores. According to normality testing, the continuous variables were expressed as means with standard deviations (SD) or medians with interquartile ranges. Inter-group comparisons were performed using the two-tailed Student’s t-test or the Mann–Whitney U test. Categorical variables were reported as counts. Comparisons between groups were made using the chi-squared or Fisher exact tests. The Spearman’s Rank correlation test was used to test the correlation of DAR and the WFNS grade. Analyses of the receiver operating characteristic (ROC) curves and the area under the ROC curves (AUC) were calculated to evaluate predictive performance. Collinearity was examined with the variance inflation factor, with the value of variables used for logistic analysis < 5. Data were analyzed using the SPSS 21.0 statistical package (SPSS Inc., Chicago, IL, USA), except for comparing ROC curves, which was performed using MedCalc statistical software version 18.9 (MedCalc Software, Mariakerke, Belgium). For all tests, *p* < 0.05 was considered statistically significant.

## 3. Results

Initially, there were 190 patients, but 12 patients were excluded for not meeting the inclusion criteria so that in the final analysis, 178 patients were included. The patient baseline characteristics are detailed in Table 1. The mean age was 58.19 ± 10.27 years and 99 patients were male. Eight had a history of diabetes mellitus and 85 had hypertension. Upon hospital admission, most patients presented with H-H classification grade of 1 or 2, and the most common WFNS grades were 1 or 2.

**Figure 1:**
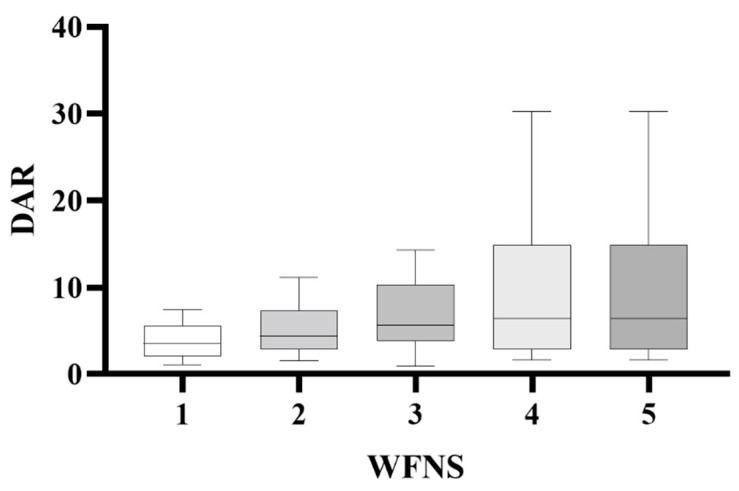
Correlation of the DAR with WFNS grade of aSAH patients on admission. Correlation of the DAR with World Federation of Neurological Surgeons Scale (WFNS) grade of aneurysmal subarachnoid hemorrhage (aSAH) patients on admission (r = 0.397, *p* < 0.001).

**Figure 2:**
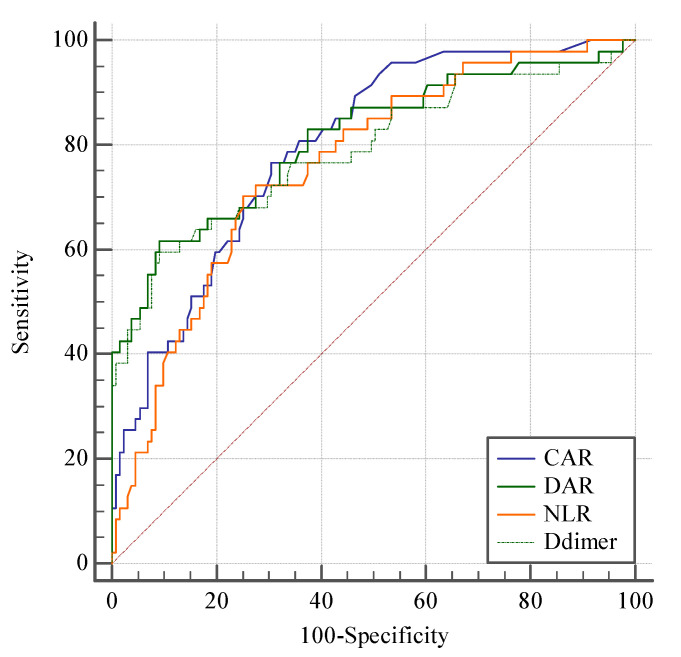
Receiver operating characteristics curves of DAR, D-dimer, CAR and NLR. Receiver operating character (ROC) curves of DAR, D-dimer, CAR, and NLR for predicting 3-month clinical outcomes in aneurysm subarachnoid hemorrhage (aSAH) patients. The area under the curve was 0.81 for DAR, 0.797 for CAR, 0.79 for D-dimer, and 0.764 for NLR.

At 3-month follow-up after aSAH, 131 patients had mRS scores of 0–2 and 47 had mRS scores of 3–6. All measured blood factors were significantly different between the good and poor outcome groups (*p* < 0.01). The good outcome group had significantly higher levels of albumin and lymphocytes than those with poor outcomes. The good outcome group had significantly lower levels of D-dimer, CRP and neutrophils than the poor outcome group. The DAR, CAR, and NLR were all significantly lower in the good outcomes group than in the poor outcomes group. DAR was significantly correlated with outcomes (OR: 1.295, *p* < 0.01). High DAR values were significantly associated with unfavorable outcomes. High WFNS grade tended to correlate with undesirable outcomes with exceptionally high odds ratios. The DAR was significantly correlated with WFNS grade at admission (Figure 1). A ROC curve was used to clarify further the predictive value of DAR for patient outcomes (Table 2). The area under the curve for DAR was greater than that of D-dimer (0.81 vs. 0.79, *p* = 0.0059), NLR (0.81 vs. 0.761, *p* = 0.368), or CAR (0.81 vs. 0.797, *p* = 0.789) (Figure 2), suggesting that DAR had a better predictive value than any single indicator or ratio.

A binary logistic regression analysis was used to analyze independent risk factors associated with prognosis in the univariate analysis. After adjustment for age, WFNS grade, CAR, DCI, and NLR, binary logistic regression identified DAR as an independent risk factor for poor 3-month outcomes in patients with aSAH (Table 3).

## 4. Discussion

Accurate outcome prediction for aSAH patients is critical for determining an appropriate therapeutic strategy. This study is the first to explore the significance of DAR in predicting aSAH outcomes. High DAR was associated with aSAH severity and outcomes after adjusting for age, WFNS grade, NLR, CAR, and DCI. These findings suggest that DAR predicts aSAH outcomes.

Hypoalbuminemia is common in patients with aSAH and is independently associated with poor outcomes [5]. We observed a negative correlation between albumin concentration and clinical outcome, consistent with former studies [12,13]. Hypoproteinemia in aSAH may be caused by systemic inflammation, malnutrition and active catabolic metabolism. Additionally, evidence suggests that albumin has neuroprotective effects via the promotion of neurovascular remodeling and attenuation of brain damage [14]. Preclinical studies have found that intravenous albumin ameliorates neurological impairment in patients with intracranial hemorrhage [14]. Furthermore, a study suggested that daily albumin treatment might have neuroprotective effects in patients with aSAH [15]. Intravenous albumin modified cerebral vascular integrity, modulated cerebral vasospasm, and mediated neuroinflammation and microglia functions [15]. These findings suggest that albumin is a promising therapy for aSAH.

D-dimer, has been investigated in diseases such as deep vein thrombosis, cerebral hemorrhage, and acute aortic dissection [16]. Elevation of D-dimer indicated enhanced fibrinolysis activity, which could be a biomarker of a hypercoagulative state or subsequent fibrinolysis in aSAH [17]. Previous studies have showed that aSAH patients with high D-dimer levels tended to have poor outcomes [4], consistent with the results reported here. Following hemorrhagic stroke, blood vessel integrity is damaged and the endogenous coagulation system is activated following tissue factor exposure. Free blood can enter the subarachnoid space, exacerbating the coagulation process and promoting microthrombosis [18], which explains the elevation of D-dimer. It has been suggested that microthrombosis is associated with blood-brain barrier (BBB) dysfunction, neuronal injury, and DCI [19]. Additionally, emerging evidence has focused on thromboinflammation—a hyper-coagulative state promoted by the occurrence of microthrombosis in response to hemorrhagic ictus or disturbed micro-circulation. this phenomenon might cause adhesion of migrating immune cells and further exaggerate inflammation, thus disrupting the integrity of the BBB, and leading to an unfavorable clinical outcome, even without occurrence of vasospasm or DCI [20].

Albumin can predict the subarachnoid hemorrhage outcome in combination with other inflammatory indices (as opposed to being used alone) [21,22]. In the present study, we used the DAR, which reflects the hypercoagulative status and malnutrition. DAR has been used as an independent prognostic marker in infections, malignancies, and other diseases [6,7], Therefore, it is not surprising that the multivariate analysis in this study revealed that DAR independently predicted poor aSAH outcomes better than D-dimer alone.

DCI caused by cerebral vasospasm is the main cause of poor prognosis after intracranial aneurysm rupture [19], and smoking is a risk factor for DCI in patients with ruptured aneurysm [23]. Elevated D-dimer was associated with cigarette smoking [24]. We evaluated the relationship between smoking and serum D-dimer based on these observations. Although we observed an elevation of serum D-dimer in the smoking aSAH group, the difference between the two groups was insignificant. Furthermore, we found no significant difference in the smoking ratio between favorable and unfavorable groups. This finding suggests that the influence of tobacco exposure on aSAH outcome was not due to increased serum D-dimer levels.

Additionally, as fibrinogen is an essential factor in fibrinolysis, we also assessed its impact on outcomes and found no significant difference between the poor and good outcome group, consistent with a previous study [25].

Other blood factors have also been assessed in aSAH patients. One characteristic feature of aSAH injury is the destruction of the BBB. The subendothelial space damaged by neutrophil infiltration plays a vital role in increasing BBB permeability. Disruption of the vessel wall can lead to leakage of plasma and molecules into the extravascular space, exacerbating cerebral edema [26]. In our study, aSAH patients with unfavorable clinical outcomes had higher neutrophil count, consistent with a previous study [27].

High CRP levels have been shown to correlate with aSAH severity. Although the underlying mechanism remains obscure, CRP could be used as a reliable prognostic factor in aSAH [28]. Previous studies demonstrated that NLR and CAR had predictive value for poor prognosis in aSAH [12,27]. In the present study, we observed a significant correlation between the predictive value of NLR and CAR on patient outcomes. We found that the predictive power of the DAR ratio was at least comparable with (if not better than) the neutrophil/lymphocyte ratio or c-reactive protein/albumin ratio. These findings suggest that DAR could be used as an independent predictor of poor aSAH outcomes.

The present study has several limitations. This is a single-center, retrospective study with limited enrollment. Additional studies with larger patient populations are warranted. The follow-up period was a short-term visit. Long-term follow-up studies are needed to validate the prognostic potential of DAR.

## 5. Conclusions

High DAR is associated with the severity of aSAH, and DAR has a significant correlation with outcomes, suggesting that DAR is a potential predictor of aSAH.

## Figures and Tables

**Table 1:** Demographic characteristics of patients.

	All Patients (178)	mRs 0–2 (131)	mRs 3–6 (47)	*p*
Sex				0.096
Male	99 (55.6%)	68 (51.9%)	31 (66%)	
Female	79 (44.4%)	63 (48.1%)	16 (34%)	
Age	58.19 ± 10.27	57.10 ± 9.48	61.23 ± 11.78	0.034
Smoking	52 (29.2%)	37 (28.8%)	15 (31.9%)	0.635
Diabetes mellitus	8 (4.5%)	6 (4.6%)	2 (4.3%)	0.644
Hypertension	85 (47.8%)	64 (48.9%)	21 (44.7%)	0.623
HH				<0.01
1	53 (29.8%)	52 (39.7%)	1 (2.1%)	
2	59 (33.1%)	56 (42.7%)	3 (6.4%)	
3	26 (14.6%)	15 (11.5%)	11 (23.4%)	
4	34 (19.1%)	7 (5.3%)	27 (57.4%)	
5	6 (3.4%)	1 (0.8%)	5 (10.6%)	
	178 (100%)	131 (100%)	47 (100%)	
WFNS				<0.01
I	63 (35.4%)	62 (47.3%)	1 (2.1%)	
II	47 (26.4%)	45 (34.4%)	2 (4.3%)	
III	18 (10.1%)	11 (8.4%)	7 (14.9%)	
IV	34 (19.1%)	12 (9.2%)	22 (46.8%)	
V	16 (9%)	1 (0.8%)	15 (31.9%)	
	178 (100%)	131 (100%)	47 (100%)	
Aneurysm location				0.69
Anterior	120 (67%)	86 (65.6%)	34 (72.3%)	
Posterior	46 (25.8%)	36 (27.5%)	10 (21.3%)	
Multiple	12 (6.7%)	9 (6.9%)	3 (6.4%)	
	178 (100%)	131 (100%)	47 (100%)	
Repair procedure (WFNS IV–V)				0.2
Clipping	28 (56%)	10 (76.9%)	18 (48.6%)	
Coiling	20 (40%)	3 (23.1%)	17 (45.9%)	
None	2 (4%)	0 (0%)	2 (5.4%)	
	50 (100%)	13 (100%)	37 (100%)	
Acute hydrocephalus	20 (11.2%)	15 (11.5%)	5 (10.6%)	0.56
DCI	9 (5.1%)	2 (1.5%)	7 (14.9%)	<0.01
Intraventricular hemorrhage	30 (16.9%)	22 (16.8%)	8 (17%)	0.566
D-dimer(ug/dL)	185.5 (98.75–324.25)	158 (87–222)	396 (203–716)	<0.01
CRP(mg/L)	8.2 (3.9–26.15)	5.7 (3.2–16.6)	25.1 (10.9–69.5)	<0.01
Alb(g/L)	39.15 (37.175–40.624)	39.6 (38.2–40.8)	36.8 (35.1–41.6)	<0.01
Fibrinogen(g/L)	2.8 (2.4–3.3)	2.8 (2.4–3.3)	2.7 (2.2–3.7)	0.524
DAR	4.868 (2.642–8.133)	4.04 (2.2–5.8)	11.03 (5.3–18.5)	<0.01
CAR	0.215 (0.0996–0.6598)	0.14 (0.09–0.41)	0.67 (0.28–1.83)	<0.01
Neutrophil(×10^9^/L)	7.9 (5.975–10.4)	7.6 (5.4–9.3)	10.2 (8.0–14.3)	<0.01
Lymphocyte (×10^9^/L)	1 (0.8–1.5)	1.1 (0.8–1.5)	0.8 (0.7–1.1)	<0.01
NLR	7.875 (4.8–12.81)	6.3 (4.31–10)	12.5 (8.36–18.2)	<0.01

Values are displayed as mean (SD), median (IQR), count (%); WFNS, World Federation of Neurological Surgeons scale; DCI, Delayed cerebral ischemia; CAR, c-reactive protein/albumin; NLR, Neutrophils/lymphocytes ratio; DAR, D-dimer/albumin.

**Table 2:** Receiver Operating Characteristic Curves for Predicting 3-month Post subarachnoid Hemorrhage Outcome.

	AUC	95%CI	Sensitivity	Specificity	*p*
CAR	0.797	0.731 to 0.854	76.6	69.5	<0.001
NLR	0.761	0.692 to 0.822	70.2	74.8	<0.001
DAR	0.81	0.745 to 0.865	61.7	90.8	<0.001
D-dimer	0.79	0.723 to 0.847	59.6	90.8	<0.001

ROC, receiver operating characteristic; CI, confidence interval.

**Table 3:** Multiple logistic regression analysis to predict poor 3-month outcome.

Object	Adjust OR	95% CI		*p*-Value
		Lower Limit	Upper Limit	
Age	0.990	0.932	1.051	0.734
DCI	2.666	0.342	20.757	0.349
DAR	1.295	1.136	1.475	<0.001
CAR	1.685	0.702	4.046	0.265
NLR	1.052	0.968	1.143	0.233
WFNS I	Ref	Ref	Ref	-
WFNS II	1.333	0.092	19.246	0.833
WFNSIII	26.544	2.522	279.399	0.006
WFNSIV-V	64.344	6.289	658.298	<0.001

CI, Confidence interval; OR, Odds ratio; WFNS, World Federation of Neurological Surgeons Scale; DCI, Delayed cerebral ischemia; CAR, c-reactive protein/albumin; NLR, Neutrophils/lymphocytes; DAR, d-dimer/albumin. *p* < 0.05 was deemed statistically significant.

## Data Availability

The datasets used and/or analyzed during the current study are available from the corresponding author on reasonable request.

## Abbreviations

aSAH	Aneurysmal subarachnoid hemorrhage
mRS	Modified Rankin Scale
WFNS	World Federation of Neurosurgical Societies
DCI	Delayed cerebral infarction
NLR	Neutrophil-to-lymphocyte ratio
CRP	C-reactive protein
CAR	C-reactive protein/albumin ratio
DAR	D-dimer/Albumin ratio
ROC	Receiver operating characteristic
